# First report of detection of microcystins in farmed mediterranean mussels *Mytilus galloprovincialis* in Thermaikos gulf in Greece

**DOI:** 10.1186/s40709-021-00139-4

**Published:** 2021-03-10

**Authors:** Maria P. Kalaitzidou, Christina I. Nannou, Dimitra A. Lambropoulou, Konstantinos V. Papageorgiou, Alexandros M. Theodoridis, Vangelis K. Economou, Ioannis A. Giantsis, Panagiotis G. Angelidis, Spyridon K. Kritas, Evanthia J. Petridou

**Affiliations:** 1 National Reference Laboratory for Marine Biotoxins, Department of Food Microbiology, Biochemical Control, Residues, Marine Biotoxins and other water toxins, Directorate of Veterinary Center of Thessaloniki, Ministry of Rural Development and Food, Limnou 3A, 54627 Thessaloniki, Greece; 2 Laboratory of Environmental Pollution Control, Department of Chemistry, Aristotle University of Thessaloniki, 54124 Thessaloniki, Greece; 3 Center for Interdisciplinary Research and Innovation (CIRI-AUTH), Balkan Center, 57001 Thessaloniki, Greece; 4 3rd Military Veterinary Hospital, General Staff, Hellenic Ministry of Defense, 15th km Thessaloniki-Vasilika, 57001 Thessaloniki, Greece; 5 Laboratory of Animal Production Economics, School of Veterinary Medicine, Faculty of Health Sciences, Aristotle University of Thessaloniki, University Campus, 54124 Thessaloniki, Greece; 6 Laboratory of Hygiene of Foods of Animal Origin-Veterinary Public Health, School of Veterinary Medicine, Faculty of Health Sciences, Aristotle University of Thessaloniki, University Campus, 54124 Thessaloniki, Greece; 7 Faculty of Agricultural Sciences, University of Western Macedonia, Florina, Greece; 8 Laboratory of Ichthyology, School of Veterinary Medicine, Faculty of Health Sciences, Aristotle University of Thessaloniki, University Campus, 54124 Thessaloniki, Greece; 9 Laboratory of Microbiology and Infectious Diseases, School of Veterinary Medicine, Faculty of Health Sciences, Aristotle University of Thessaloniki, University Campus, 54124 Thessaloniki, Greece

**Keywords:** Microcystins, *Mytilus galloprovincialis*, cyanobacteria, Thermaikos gulf, ELISA, Mass spectrometry

## Abstract

**Background:**

Microcystins are emerging marine biotoxins, produced by potentially toxic cyanobacteria. Their presence has been reported in aquatic animals in Greek freshwater, while data are few in marine environments. Since the climate change induces eutrophication and harmful algal blooms in coastal marine ecosystems affecting the public health, further research on microcystins’ presence in marine waters is required. The aim of this study was to examine the potential presence of microcystins in mussels *Mytilus galloprovincialis* in the largest farming areas in Thermaikos gulf, in Northern Greece, and to investigate their temporal and spatial distribution, adding to the knowledge of microcystins presence in Greek Mediterranean mussels.

**Results:**

A 4-year microcystins’ assessment was conducted from 2013 to 2016, in farmed Mediterranean mussels *M. galloprovincialis*, in five sampling areas in Thermaikos gulf, in northern Greece, where the 90% of the Greek mussels’ farming activities is located. The isolation of potentially toxic cyanobacteria was confirmed by molecular methods. An initial screening was performed with a qualitative and quantitative direct monoclonal (DM) ELISA and results above 1 ng g^−1^ were confirmed for the occurrence of the most common microcystins-RR, -LR and -YR, by Ultra High Performance Liquid Chromatography (UHPLC) coupled with a high- resolution mass spectrometer (HRMS) (Orbitrap analyzer). Microcystin-RR and microcystin-LR were detected, while the intensity of microcystin-YR was below the method detection limit. Most samples that exhibited concentrations above 1 ng g^-1^ were detected during the warm seasons of the year and especially in spring. Results indicated an overestimation of the ELISA method, since concentrations ranged between 0.70 ± 0.15 ng g^−1^ and 53.90 ± 3.18 ng g^−1^, while the confirmation denoted that the levels of microcystins were 6 to 22 times lower.

**Conclusions:**

Microcystin-RR and microcystin-LR were detected for the first time in mussel *M. galloprovincialis*, harvested from farms in Thermaikos gulf, in Central Macedonia, Greece. Their presence was linked to potentially toxic cyanobacteria. Bioaccumulation was observed in digestive gland, while the concentrations in muscles were found extremely low. Samples with levels above 1 ng g^−1^ were observed mostly during spring, confirming the seasonal distribution of microcystins. The comparison of the results by the ELISA and the LC-Orbitrap MS method indicated an overestimation of concentration by the ELISA method.

## Background

Global climate change, nutrient enrichment of water bodies and eutrophication are among the responsible factors that have induced cyanobacterial blooms worldwide [1]. The abundance of potential toxic cyanobacteria species is prone to the production of secondary metabolites, the cyanotoxins. Microcystins (MCs) are the most common group of cyanotoxins, with high toxicity [2] and they are considered as emerging toxins [3]. They are produced by different genera of freshwater cyanobacteria, such as *Microcystis, Anabaena (Dolichospermum), Nostoc, Planktothrix, Chroococcus* [4], as well as by marine picoplanktonic species, such as *Synechococcus* and *Synechocystis* [5]. Microcystins are monocyclic heptapeptides, having a general structure of cyclo-(D-alanine-R1-DMeAsp-R2-ADDA-D-glutamate-Mdha), where R1 and R2 are variable L-amino acids [1]. Up to date, more than 279 congeners have been recognized [2]. ADDA, [(2 S,3 S,8 S,9 S)-3-amino-9-methoxy-2,6,8-trimethyl-10-phenyldeca-4,6-dienoic acid], is a unique amino acid, reported only in microcystins and in a similar group of pentapeptides, the nodularins [6]. The presence of ADDA defines the toxicity of microcystins [7]. Microcystins toxic effects have been reported in humans, domestic and wild animal and in aquatic organisms [7]. The inhibition of the protein phosphatases PP1 and PP2A, has been described as the primary mechanism of toxicity expression, inducing hepatotoxicity, apoptosis, and necrosis of the hepatocytes [2, 8]. Moreover, microcystins have been related to toxic effects in other organs, such as heart [9], kidney [2], intestine [10], lung [11] and brain [12]. Microcystin-LR (MC-LR) is considered the most common analogue with the highest toxicity, while microcystin-RR (MC-RR) and microcystin-YR (MC-YR) follow [13]. Moreover, it is reported that chronic exposure to MC-LR can lead to primary liver cancer [14, 15], and it is considered as potential carcinogenic (group 2B) [16]. The detection of microcystins has been reported mainly in freshwater, as well as in brackish and marine environments [1]. Moreover, their presence in food web and especially in seafood such as shellfish, fish and crustaceans is considered hazardous for the public health [1]. In Greece, the detection of microcystins have been reported in the lake fish *Cyprinus carpio*, freshwater mussel *Anodonta* sp., in the freshwater European crayfish *Astacus astacus* and in the amphibian *Rana epirotica* skin [17, 18]. Regarding the presence of microcystins in marine aquatic organisms, there is only one report in Mediterranean mussels *Mytilus galloprovincialis* from Amvrakikos gulf, a closed embayment in Ionian Sea [5]. Exports of shellfish and especially *M. galloprovincialis* mussels, contribute significantly to Greek economy. The main farming activity (almost 90%) of *M. galloprovincialis*, is practiced in the Thermaikos gulf areas of Chalastra, Imathia and Pieria in northern Greece [19]. Thermaikos is a semi-closed gulf (90 m maximum depth, surface of 5100 km^2^), located in the north-west Aegean Sea, in Central Macedonia Greece. It is enriched by four rivers (Axios, Loudias, Aliakmonas and Gallikos), often inducing eutrophication and harmful algal blooms [20, 21]. In this study two methods were applied to detect microcystins in farmed mussels of Thermaikos gulf, ELISA and mass spectrometry. The aim of the study was to investigate the temporal and spatial distribution of the microcystins in mussels *M. galloprovincialis*, in these mussels’ farming areas, as well as to compare the aforementioned detection methods proposed by the scientific literature. The results would contribute to the knowledge of microcystins presence in Greek mussels, to the proposed methodology and to protection of public health.

## Methods

### Sampling area

**Fig. 1:**
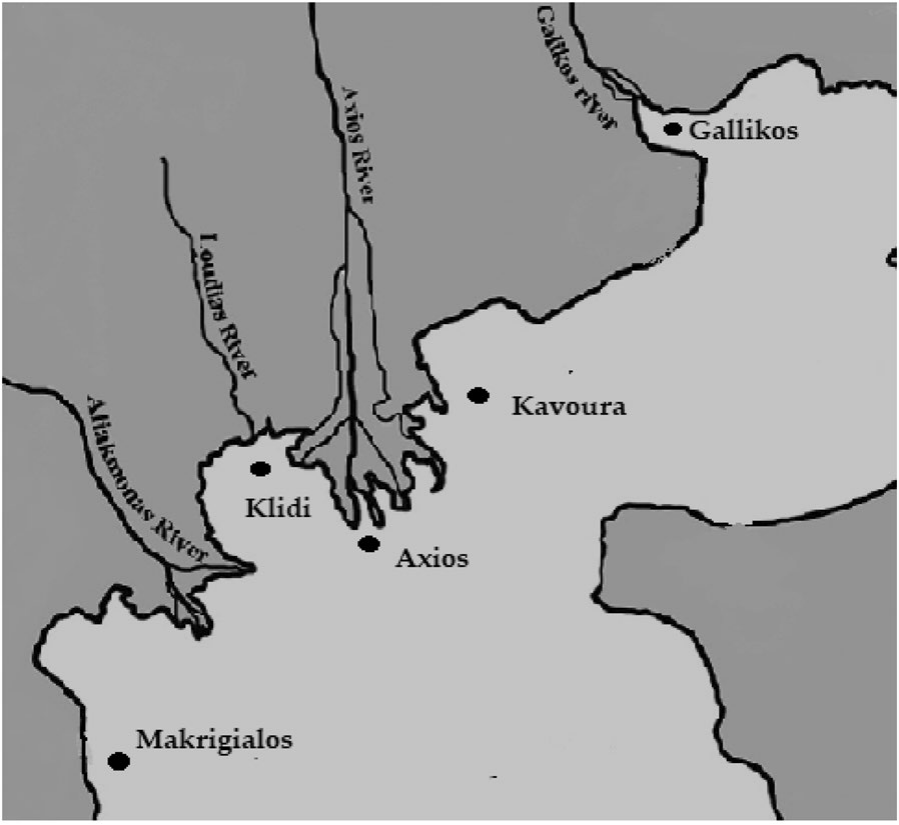
Sampling area of the study, in Thermaikos gulf, in Central Macedonia

In this study, five sampling sites in Thermaikos gulf had been used for *M. galloprovincialis* sampling, namely Kavoura Chalastra (40° 32′ 20.12″ N, 22° 44′ 56.63″ E), Klidi Imathia (40° 28′ 37.03″ N, 22° 39′ 58.94″ E), Makrigialos Pieria (40° 24′ 57.98″ N, 22° 37′ 14.93″ E), Delta of Axios river (40° 31′ 11.24″ Ν, 22° 45′ 44.33″ Ε) and estuaries of Gallikos river (40° 37′ 50.85″ N, 22° 50′ 46.13″ E). These sites had been selected on the basis of their high economic and environmental importance, as well as being potential source of pollution by industrial, agricultural, veterinary, and urban wastes (Fig. 1). They included *M. galloprovincialis* growing farms employing the “long line farming system” up to the age of 15 to 18 months, e.g. until mussels reach a commercial size of at least 5 cm. During a 4-year period (2013–2016), a total of 750 sample batches had been seasonally collected approximately as follows: 55 sample batches during each spring, 55 sample batches during each summer, 52 sample batches during each autumn, and 25 sample batches during each winter (winter samples derived from two areas only due to seasonal reduction of production). Each sample batch consisted of 150 to 300 mussels (smaller mussels during winter) and weighed approximately 1.5 to 2.0 kg. During the sampling, abiotic parameters of the water (temperature, salinity, pH and dissolved oxygen) were measured, using a handheld multiparameter instrument (YSI 556, YSI Incorporated, Ohio, USA). The samples were transported in a portable refrigerator at 4 ± 1 °C and forwarded within 4 h to the Laboratory of Microbiology and Infectious Diseases, Faculty of Veterinary Medicine, Aristotle University of Thessaloniki. Before processing the shell was cleaned and dead mussels were discarded.

### Chemicals and reagents

Methanol water and acetonitrile of LC-MS grade, as well as formic acid 99–100% a.r. were purchased from Sigma Aldrich Co. (St. Luis, U.S.A.). HLB OASIS cartridges were used for Solid Phase Extraction (Waters, Milford, MA, USA). For the molecular detection of cyanobacteria QIAamp DNA Mini Kit was purchased by Qiagen (Qiagen, USA), MyTaq™ Red Mix from BIOLINE (Bioline, UK) and NanoDrop (Shimadzu, Japan) was used for the evaluation of the quality and quantity of the extracted DNA. For screening a direct competitive microcystins (ADDA)-DM (direct monoclonal) ELISA kit was purchased from CD Creative Diagnostics (New York, USA). Certified reference materials (CRMs) of microcystin-LR, -RR and -YR were purchased from CIFGA (Lugo, Spain) and uncontaminated mussels’ tissue (CRM ZERO MUS) from National Research Council Canada (Newland and Labrador, Canada). The LC separation of the microcystins was carried out on a C18 reverse-phase column ACCLAIM POLAR ADVANTAGE II 3 μm 3 × 150 mm with guard column 2/pk and holder-coupler.

### Sample preparation

From each sample batch, 50 to 60 live mussels with mean length of 5.9 ± 0.50 cm and mean body weight 6.1 ± 0.64 g were cleaned and deshelled. Their soft interiors were split either in a digestive gland pool or in a muscle pool (each pool was weighing approx. 150 g). Each pool was placed in a sieve to drain, homogenized and 5 ± 0.1 g were transferred in a 50 mL polypropylene centrifuge tube. An aqueous solution of methanol 75% (75:25 v/v methanol:ultrapure water) was used to extract the microcystins from the soft tissues of the mussels, since it is proposed as the most suitable solvent for the recovery of the toxins under investigation [22–24]. After the extraction, a clean-up procedure using solid phase extraction (SPE) was performed [25]. The extracts were evaporated at a final volume of 2 mL. The clean-up procedure was performed in a vacuum manifold system (Alltech® Vacuum Manifold, 2051 Waukegan Rd, Bannockburn, U.S.A), using OASIS HLB (Hydrophilic-Lipophilic Balance) cartridges (200 mg per 6 cc). After the elution, 0.5 mL from the fraction was received and dried under nitrogen stream, reconstituted with 2 mL Milli-Q water and analyzed by ELISA. The rest of the cleaned extraction was used for the confirmation by Ultra High Performance Liquid Chromatography–tandem mass spectrometry.

### Microcystins analysis

#### Detection by ELISA

Initially screening was performed by ELISA [26–28]. Among several commercial kits, a direct competitive ELISA for microcystins and nodularins, ([(2 S,3 S,4E,6E,8 S,9 S)- 3-amino-9-methoxy-2,6,8- trimethyl-10-phenyldeca-4,6-dienoic acid, ADDA)-direct monoclonal (DM) ELISA, was chosen [29]. The analysis was performed in triplicate according to the manufacturer’s instructions (CD Creative Diagnostics, New York, USA). The reproducibility and the recovery were evaluated by the analysis of a spiked sample of uncontaminated mussel tissue with microcystin-LR (2 µg g^−1^). The analysis of the spiked sample was repeated ten times and the obtained recovery was 89%. The limit of detection (LOD) according to manufacturer was set at 0.1 ng g^−1^ of microcystin-LR. All standards (0.15 ng g^−1^ to 5 ng g^−1^) were measured in duplicate, as well as control (0.75 ± 0.185 ng g^−1^), calibrator (standard 0) and the spiked sample. The absorbances in the microplate of the kit were measured at 450 nm with a spectrophotometer (model A3, DAS, Rome, Italy). A standard curve was used to calculate the values and the results were expressed as microcystin-LR equivalents (µg kg^−1^ wet weight). Samples with concentrations higher than 1 ng g^−1^ were further analyzed by LC-Orbitrap HRMS.

#### Confirmation by LC-Orbitrap HRMS

The confirmation of the ELISA results was obtained by using certified standards of microcystin-RR (MC-RR), microcystin-LR (MC-LR) and microcystin-YR (MC-YR). The analysis was performed using a high-resolution Q Exactive Focus Orbitrap mass spectrometer equipped with a heated electrospray ion source (HESI) operating in positive ionization mode, coupled to an Ultimate 3000 ultra-high-performance liquid chromatography system (Thermo Scientific, USA). A reverse phase C18 column Acclaim Polar Advantage II 3 µm^3^ × 150 mm in conjunction with a guard column 2/pk and a Holder-Coupler was used to separate the analytes (Thermo scientific, USA). The mobile phases were water (A) and acetonitrile (B), both acidified with 0.1% formic acid. The chromatographic conditions were the following: column temperature 40 °C, injection volume 20 µL, flow rate 0.3 mL min^−1^ and total run time 11 min. The gradient elution program is shown in Table 1.

**Table 1:** Gradient elution program during the chromatographic analysis

Time	Mobile phase A%	Mobile phase B%
0.0		
0.0	98.0	2.0
0.1	98.0	2.0
1.5	98.0	2.0
2.0	80.0	20.0
3.0	60.0	40.0
7.4	40.0	60.0
7.5	2.0	98.0
8.0	2.0	98.0
9.0	98.0	2.0
11.0	98.0	2.0

The HESI source parameters were the following: capillary temperature 320 °C, spray voltage 3.5 kV, auxiliary gas heater 413 °C, sheath gas flow rate 48 au, auxiliary gas flow rate 11 au, and sweep gas flow rate 2 au. The S-lens RF level was set at 50.0. A full-scan MS acquisition was carried out in a mass range of 400–1100 (*m/z*). The resolution was set at the maximum available value (70,000) and the spectrum data were obtained using the centroid algorithm. The maximum injection time (IT) was set to auto and the AGC target (automated gain control) to 10^6^ ions. For the ddMS2 (data-dependent) fragmentation, a stepped collision energy (30, 50, 70 eV) was applied. The instrumental limit of quantification (LOQ) was 0.017 µg L^−1^ for microcystin-RR, 0.084 µg L^−1^ for microcystin-LR and 1.495 µg L^−1^ for microcystin-YR. Raw data were processed with the aid of Xcalibur software, version 4.1 (Thermo scientific, USA) and the results were reported as total microcystin concentration (µg kg^−1^ wet weight).

### Molecular detection of cyanobacteria species

The potentially toxic cyanobacteria that were cultured from water samples of the sampling areas as described earlier [30], were subjected in DNA isolation using the QIAamp DNA Mini Kit (Qiagen, USA) following the manufacturer’s protocol. The quality and quantity of the extracted DNA was evaluated in a NanoDrop spectrophotometer (Shimadzu, Japan). Approximately 50 ng of extracted DNA were utilized in a PCR using the MyTaq™ Red Mix (Bioline, UK) and the modified primers 27f-CM and 1492r [31] that amplify a part of the 16Sr RNA bacterial genome. PCR regime was as follows: after an initial desaturation step of 3 min at 95 °C, 38 cycles of 30 s at 95 °C, 40 s at 51 °C and 50 s at 72 °C were applied, followed by a final extension step of 7 min at 72 °C. PCR products were visualized after electrophoresis in 1.5% agarose gel stained with 0.5 µg ethidium bromide (Invitrogen™) per ml.

### Statistical analysis

Both parametric and non-parametric statistical methods were applied for the statistical evaluation of the data. The assumptions of normality and homogeneity of variances for the microcystins concentrations of digestive gland were tested using the Shapiro Wilk and Levene’s test, respectively. In case of normality and variances homogeneity, one way ANOVA was performed to evaluate possible mean effects of sampling sites and season on the microcystins concentrations, while differences between mean values of specific groups were evaluated using the Duncan’s new multiple range tests. In cases where the assumptions of variability and/or normality of the distributions were seriously violated, the Kruskal-Wallis non-parametric test was applied to evaluate group differences, while differences between specific groups were evaluated using the Mann-Whitney U-test. All analysis were conducted using SPSS version 25 software (IBM Inc., Armonk, NY, USA). Significance was declared at *p*-value < 0.05.

## Results

Microcystins above 1 ng g^−1^ were detected by ELISA in 52 samples out of 750. On the other hand, only 15 of them were confirmed by LC-Orbitrap HRMS. The identification was based on the presence of at least one peak corresponding to the pseudomolecular ion of the microcystin, at a retention time (t_*R*_) matching to that of the certified standards with a drift < 0.2 min, as well as the determination of the exact mass with a mass error (Δ) < 5 ppm. The number of samples above 1 ng g^−1^ for both methods are shown in Table 2. No microcystins were detected during winter (levels lower than the LOD of both methods). Moreover, concentrations higher than 1 ng g^−1^ were detected only at the digestive gland samples; on the contrary, all muscle samples were below the limit of detection. Also, the presence of cyanobacteria was confirmed molecularly, by the visualization of one clear band of approximately 1400 bp in size in all tested samples. The quantitative and qualitative analysis with ELISA showed statistically significant difference between areas at the same season and also in the same area in different seasons. The mean values of the concentrations of microcystins obtained during the screening were up to 53.02 ± 1.09 ng g^1^ in spring (Delta of Axios river), 45.05 ± 1.20 ng g^−1^ in summer (Klidi) and 53.90 ± 3.18 ng g^−1^ in autumn (Makrigialos). Regarding the same area at different seasons, the higher concentrations in Kavoura (Chalastra) were detected during spring (28.60 ± 2.32 ng g^−1^), in Makrigialos the lowest concentration of microcystins were detected in summer (16.70 ± 2.49 ng g^−1^) and in Klidi in autumn (36.28 ± 2.21 ng g^−1^). At the sampling areas at the estuaries of Axios and Gallikos rivers, the highest concentrations were detected during spring, i.e. 53.02 ± 1.09 ng g^−1^ and 20.4 ± 3.07 ng g^−1^, respectively. The temporal and spatial distributions of microcystins concentrations are given in Table 3.

**Table 2:** Temporal and spatial distribution of microcystins in farmed mussels in the sampling areas in Thermaikos gulf, analyzed by ELISA and LC-Orbitrap HRM

Sampling area	Spring		Summer			Autumn		Winter	
	ELISA	LC-MS	ELISA		LC-MS	ELISA	LC-MS	ELISA	LC-MS
Kavoura	7/60	2/60	4/60		1/60	2/60	Nd	Nd	Nd
Makrigialos	7/60	1/60	5/60		1/60	5/60	2/60	Nd	Nd
Klidi	3/60	1/60	3/60		2/60	5/50	1/50	Nd	Nd
Gallikos river estuaries	3/20	Nd	Nd		Nd	2/20	Nd	Nd	Nd
Axios river estuaries	4/20	3/20	Nd		Nd	2/20	1/20	Nd	Nd
Total	24/220 (10.9%)	7/220 (3.2%)	12/220 (5.5%)		4/220 (1.8%)	16/210 (7.6%)	4/210 (1.9%)		

*Nd* not detected

Numerators declare the number of the samples, where microcystins above 1 ng g^−1^ were detected

Denominators declare the total number of the samples per area and season

**Table 3:** ELISA screening. Temporal and spatial distribution of microcystin concentrations (mean ± SD, ng g^−1^) of digestive gland in farmed mussels of Thermaikos gulf

Season	Sampling area				
	Kavoura	Makrigialos	Imathia	Gallikos estuaries	Axios estuaries
Spring	28.6 ± 2.32^Aa^	51.32 ± 1.010^Ab^	51.8 ± 1.44^Ab^	20.4 ± 3.07^Ac^	53.02 ± 1.090^Ab^
Summer	18.9 ± 2.18^Aa^	16.7 ± 2.49^Ba^	45.05 ± 1.200^Ab^	Nd	Nd
Autumn	1.23 ± 0.98^Ba^	53.9 ± 3.18^Ab^	36.28 ± 2.210^Bc^	1.4 ± 0.82^Ba^	1.5 ± 0.73^Ba^
Total	20.2 ± 4.70^a^	42.9 ± 9.47^b^	48.7 ± 2.51^b^	9.5 ± 5.13^c^	22.6 ± 1.85^a^

*SD* standard deviation

Values with different small superscript in the same row indicate difference at the 5% significance level (parametric tests were applied)

Values with different capital superscript in the same column indicate difference at the 5% significance level (Non parametric tests were applied)

Total sample size per sampling site: N = 180 in Kavoura, N = 180 in Makrigialos, N = 170 in Klidi, N = 60 in Axios estuaries, N = 60 in Gallikos estuaries

Total sample size per season: N = 220 in spring, N = 220 in summer, N = 210 in autumn

**Fig. 2:**
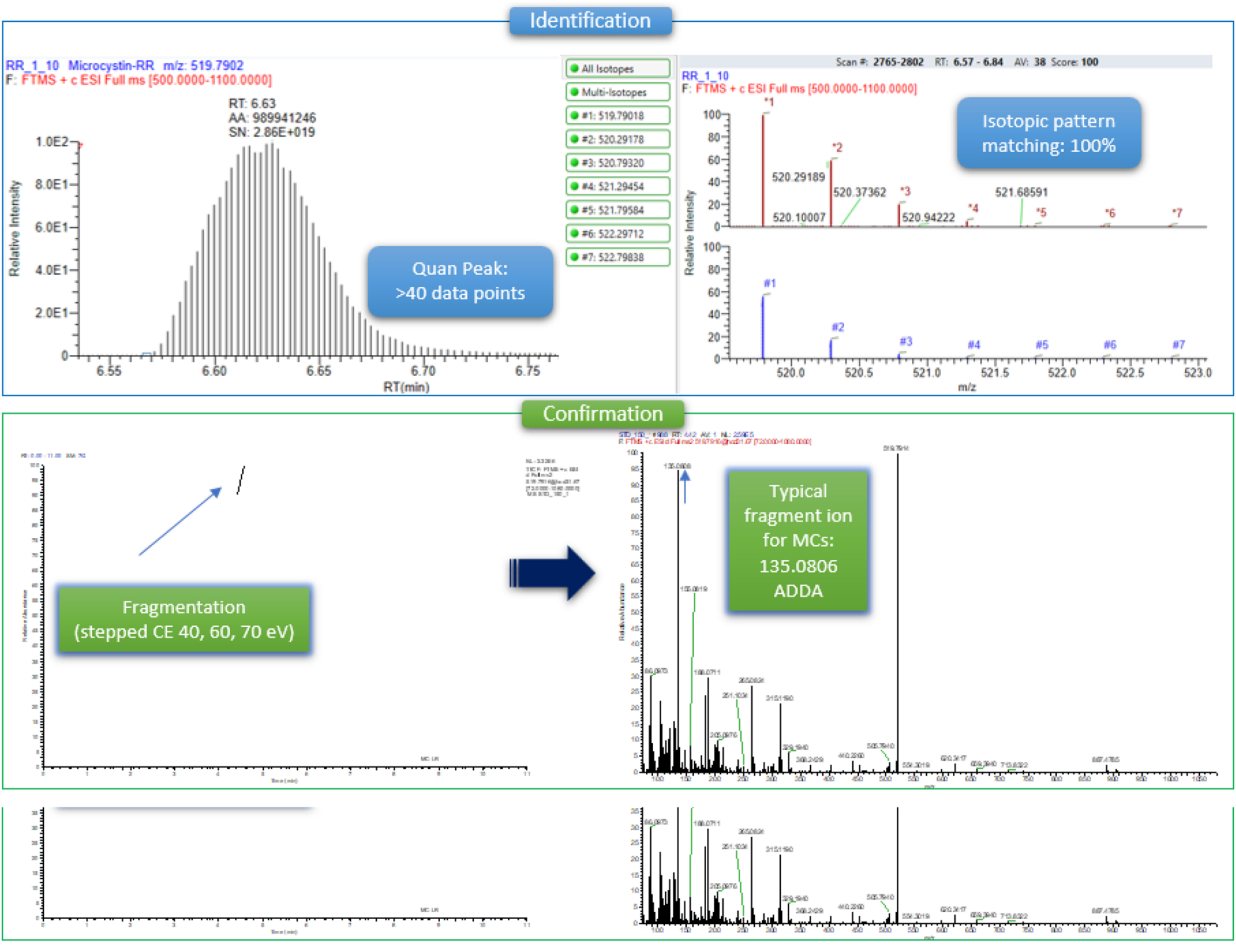
Visualization of the applied steps for the identification and confirmation of microcystin-RR

**Fig. 3:**
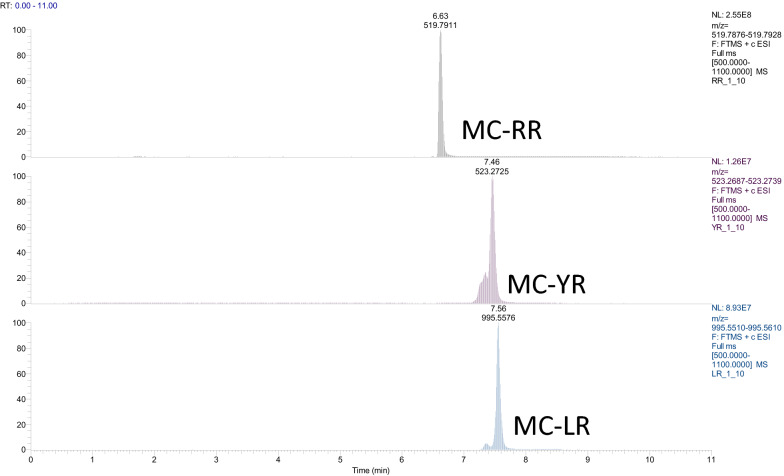
Extracted ion chromatograms of a standard mixture containing microcystin-RR, -RL and -YR (certified reference material) at a concentration of 50 µg L^−1^

**Fig. 4:**
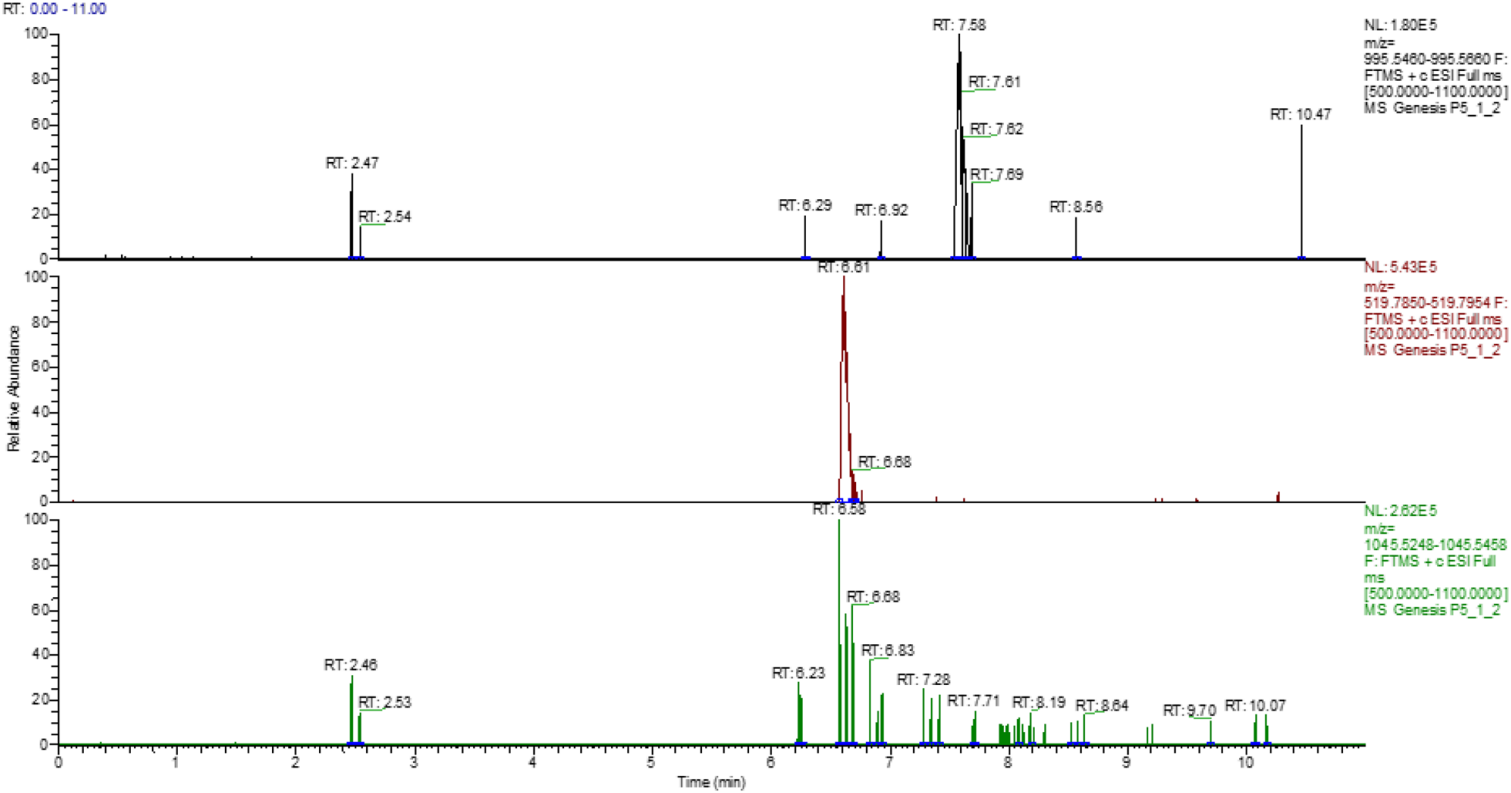
Chromatogram of farmed mussel near the estuaries of Delta of Axios river. Retention time of microcystin-LR is 7.58 min and of microcystin-RR 6.61 min

**Fig. 5:**
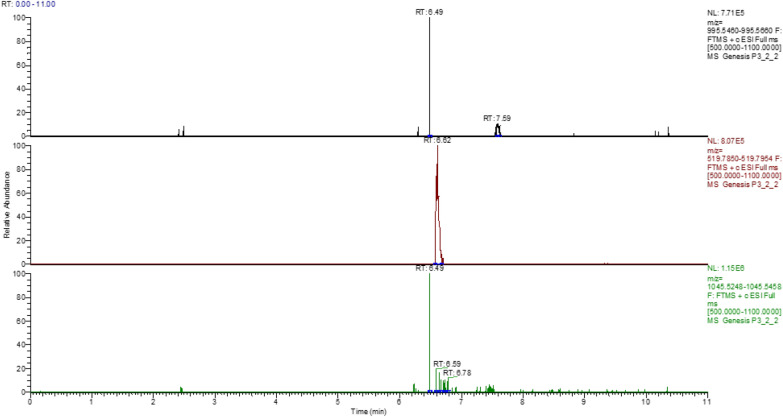
Chromatogram of farmed mussel in the area of Imathia. Retention time of microcystin-LR is 7.9 min and of microcystin-RR 6.61 min

 The microcystins that were identified by LC-Orbitrap HRMS were microcystin-RR and microcystin-LR in traces. The identification of the compounds was based on the detection of the molecular ion(s) that were the monocharged and bicharged ions of the initial molecule (Table 4) as well as the isotopic pattern matching, while the confirmation was based on the existence of a characteristic fragment. In the case of microcystins, the typical fragment corresponds to ADDA, with a theoretical accurate mass 135.0806. Figure 2 represents the steps for the identification and confirmation. It is noteworthy that the toxicity of microcystins is mainly attributed to the presence of the uncommon amino acid ADDA. No microcystin-YR was detected in all samples. The (t_*R*_) of the certified standards were 6.63 min for MC-RR, 7.56 min for MC-LR and 7.46 min for MC-YR (Table 4). Figure 3 shows the chromatograms of the certified standards, used in the method. Concentration of the confirmed samples were lower than the ones found by ELISA (Table 5). The highest levels were observed in mussels harvested near the Delta of Axios river in May and in Klidi (Imathia) in June (Figs. 4 and 5). In the first case concentrations in the digestive gland were 6.25 ng g^−1^ of MC-RR and 1.35 ng g^−1^ of MC-LR and in the second 1.08 ng g^−1^ of MC-RR and 0.15 ng g^−1^ of MC-LR, respectively. Regarding the comparison between the two methods, an over estimation was observed by ELISA.

**Table 4:** LC-Orbitrap MS/MS data for the studied microcystins

Microcystins	Molecular Formula	t*R* (min)	Monocharged ion [M+H]^+^				Bicharged ion [M+H]^2+^			
			Theor. accurate mass	Exp. accurate Mass	Δ (ppm)	RDBE	Theor. accurate mass	Exp. accurate mass	Δ (ppm)	RDBE
MC-RR	C_49_H_75_N_13_O_12_	6.63	1038.5731	1038.5743	1.1554	17.5	**519.7902**	519.7910	1.609	18
Fragment	C_9_H_11_O						135.0806	135.0807	0.7403	4.5
MC-LR	C_49_H_74_N_10_O_12_	7.56	**995.5560**	995.5572	1.151	17.5	498.28166	498.28170	0.0803	17
Fragment	C_9_H_11_O		135.0806	135.0808	1.4806	4.5				
MC-YR	C_52_H_72_N_10_O_13_	7.46	**1045.5353**	1045.5372	1.8380	21.5	523.2713	523.2725	0.5813	21
Fragment	C_9_H_11_O		135.0806	135.0808	1.4806	4.5				

Ions from which the fragments were generated are shown in bold

**Table 5:** Microcystins concentration in the digestive gland and in the flesh of farmed mussels *Mytilus galloprovincialis* in Thermaikos gulf

Sampling areas	Concentrations	
	Digestive gland	Flesh
	Mean ± SD (ng g^−1^)	Mean ± SD (ng g^−1^)
Kavoura	1.30 ± 4.22^a^	Nd
Makrigialos	1.00 ± 3.88^a^	Nd
Klidi	1.05 ± 3.950^a^	Nd
Gallikos estuaries	0.13 ± 0.016^b^	Nd
Axios estuaries	7.60 ± 0.58^c^	Nd

*Nd* not detected at levels higher than limit of detection (LOD)

*SD* standard deviation

Values with different superscript indicate difference at the 5% significance level

## Discussion

The obtained results revealed the presence of microcystins in farmed mussels *M. galloprovincialis*, in Thermaikos gulf for the first time, while concentrations above 1 ng g^−1^ were found in digestive gland. Microcystins in mussels and other seafood have been detected globally [32]. Microcystins-LR and -RR are considered the most common [33], while MC-LR has been described as the toxin most detected in mussels, with MC-RR and MC-YR following [13, 34]. In a survey in freshwater bivalves *Sinanodonta woodiana, Sinanodonta arcaeformis*, and *Unio douglasiae* from a wetland in South Korea [35], it was found that the concentrations of MC-RR, MC-LR and MC-ΥR, were 11.2 to 70.1 µg g^−1^ dry weight in the muscles and 168.9 to 869 ng g^−1^ dry weight in the digestive gland. The same microcystins were detected in a similar study in freshwater mussels (*Cristaria plicata, Hyriopsis cumingii* and *Lamprotula leai*) in lake Taihu in China [36], at high concentrations mostly in the digestive gland, reaching up to 38478.1 ng g^−1^ dry weight. Also, MC-RR, MC-LR and MC-YR were detected for the first time in lake Dau Tieng in Vietnam, in mussels *Corbicula* sp. and *Ensidens* sp. at concentrations 1.54 ± 0.21 and 3.15 ± 0.65 µg MC g^−1^ dry weight, respectively [37]. Another study in North Baltic Sea, showed that the blue mussels *Mytilus edulis* examined by ELISA, accumulated microcystins up to 2150 ± 60 ng g^−1^, expressed as equivalents of microcystins and nodularins. At the same study it was reported that these toxins were detected in the liver of the fish *Platichthys flesus*, at concentrations up to 99 ± 5 ng g^−1^; hence no toxins were detected in the muscle [38]. A similar study [39] in mussels harvested in the North-East Pacific Ocean of British Columbia, reports the presence of MC-LR at levels up to 600 ng g^−1^, as measured by LC/MS, and also in mussels imported from Canada and the Netherlands.

Microcystins in Greece have been detected mostly in freshwater mussels linked to the occurrence of cyanobacterial blooms [5, 17]. In the freshwater mussel *Anotonda* sp. in Lake Kastoria, microcystins have been detected at the level of 3271 ng g^−1^ equivalents of MC-LR dry weight, by the protein phosphatase 1 inhibition assay test (PP1IA) [17]. Regarding the Mediterranean mussels *M. galloprovincialis*, there is only one report in Amvrakikos gulf, in Western Greece, where microcystins were detected after a *Synechococcus* sp. and *Synechocystis* sp. bloom [5]. The concentrations of the toxins were 45 ± 2 to 141.5 ± 13.5 ng g^−1^ equivalents of MC-LR (ELISA), values that according to the researchers were above the Tolerable Daily Intake (TDI), as it is set by the World Health Organization (WHO).

In Greece, microcystins have been also detected in other aquatic animal tissues. A research in fish from the lakes Kastoria, Iliki, Kerkini and Pamvotis, and Gallikos river, showed that microcystins were accumulated in the fish *Cyprinus carpio*, *Carassius gibelio*, *Silurus aristotelis* and *Perca fluviatilis* and in the amphibian *Rana epirotica*. The samples were analyzed by ELISA and the toxins concentrations were 20 to 1440 ng g^−1^ dry weight in the muscles and 25 to 5400 ng g^−1^ dry weight in visceral tissue [17]. Microcystins were also detected in fish *Carassius gibelio* in lake Pamvotis. It was reported that the toxins were accumulated mostly in liver samples (124.4 ng g^−1^) and less in muscle samples (7.1 ± 2.5 ng g^−1^), analyzed by ELISA [40]. Similar study was contacted in *Cyprinus carpio* tissues in lake Karla in Thessaly. It was observed that the highest concentrations were accumulated in liver (732 ± 350 ng g^−1^), while kidneys (362 ± 207 ng g^−1^) and muscles (362 ± 207 ng g^−1^) were following [41].

Our study indicates that microcystins can be bioaccumulated in mussels, a finding that is in accordance with the literature [42–46]. The fact that mussels are filter feeders, enables them to bioaccumulate environmental pollutants [47], like microcystins [48]. A study in the brackish waters of Curonian lagoon [49] in Lithuania, revealed that mussels *Dreissena polymorpha* (zebra mussels), accumulated microcystins at high concentrations, up to 139 ng g^−1^ dry weight analyzed by ELISA and 284 ng g^− ^ dry weight by PPIA. The toxins were detected even at low abundances of potentially toxic cyanobacteria periods. It was assumed that the bioaccumulation in mussels could be explained by a secondary contamination by resuspended microcystins residues in sediment particles [48]. Moreover, cyanobacteria are considered food for mussels, leading to the uptake of intracellular cyanotoxins while it remains unclear, whether this takes place via the uptake of dissolved or intracellular hepatotoxins [38].

According to our study, the target organ of microcystins bioaccumulation seems to be the digestive gland. Several studies have reported the bioaccumulation of microcystins in the digestive gland of freshwater, brackish and marine mussels, at concentrations higher than of other organs [35, 36, 42, 43]. Moreover, during an experimental contamination, pearl mussels (*Hyriopsis cumingii*) from an aquaculture of Yueshan village, Ezhou city, in China, were exposed to *Microcystis aeruginosa* 905 for 15 days. After the analysis by HPLC-UV, it was found that MC-LR was accumulated in hepatopancreas at the highest level of 55.78 ± 6.73 µg g^−1^ dry weight, while the concentrations in other organs were 27.88 ± 2.22 µg g^−1^ dry weight in gonads, 5.66 ± 0.55 µg g^−1^ dry weight in gills and 5.17 ± 0.87 µg g^−1^ dry weight in muscles [50].

 Except from bioaccumulation, the biomagnification of microcystins in aquatic animals has been a field of study for many researchers, leading to different opinions. According to some studies, no biomagnification is observed, or it is not documented sufficiently [36, 37, 51, 52]. Moreover, biomagnification has been observed more in lipophilic marine biotoxins rather than in hydrophilic ones, as microcystins [48]. On the other hand, biomagnification was reported in farmed and wild bivalves (mussels, oysters and clams), where concentrations of microcystins up to 107 times higher than the ones in water were reported [53]. Also, it has been reported that biomagnification of cyanotoxins in aquatic animals could be explained by their evolution in relation to cyanobacteria [54], leading to their ability to develop defense mechanisms against the cyanotoxins [55]. Through this the aquatic animals could bioaccumulate toxins at high levels and thus be carriers in the food chain, contributing to biomagnification. In a recent meta-analysis of field studies, biomagnification and biodilution of microcystins in aquatic foodwebs (zooplankton, mollusks, fish, decapods, turtles and birds) was assessed [56]. The biomagnification factor (BMF) for microcystins was calculated by the researchers as the ratio between their concentration detected in those organisms and their diet. Biodilution was sufficient. Zooplankton showed potential for microcystins biomagnification, influencing their concentrations in the liver of zooplanktivorous fishes and carnivorous jellyfish, where high values of BMF were observed. There seemed to be a proportional increase between the duration of consumers exposure to diet, having high microcystins concentrations. It was concluded that the exposure to high populations of potential toxic cyanobacteria of aquatic animals, especially those that are part of the human food chain, could be related to biomagnification.

In our study, results above 1 ng g^−1^ were observed mostly during warm seasons and especially in May. This could be explained by the increase of sunlight, the high temperatures in the water and the enrichment of Thermaikos gulf with nutrients from the adjacent rivers. A survey in Greek internal waters showed that the highest levels of MC-LR were observed during the warm months of the year [57]. Microcystin-RR, and microcystin-LR have also been detected at 79.4% and 73.5%, respectively in lakes in Greece during warm seasons [17]. In another study by ELISA in lake Koronia, microcystins were detected at higher levels during spring [58]. Moreover, mussels *M. galloprovincialis* in Amvrakikos gulf accumulated the highest concentrations of microcystins in the same season [5]. Same findings have been reported in other Mediterranean countries. In Italy, a survey in lakes Garda, Como, Iseo, Lugano and Maggiore revealed that the highest concentrations of microcystins and anatoxin-a were observed during warm seasons and mostly in May and September [59]. Additionally, in South-East Adriatic, in mussels *M. galloprovincialis* analyzed by ELISA, microcystins were detected up to 256 ng g^−1^. Moreover, they were detected up to 2.3 ng g^−1^ in clams *Chamelea gallina* [60]. A similar survey in Portuguese recreational waters showed that the highest levels of microcystins were recorded during spring and summer, and mostly from April to September [61]. Moreover, it has been reported that the global climate change and the increase of the temperature in water environments induce potentially toxic cyanobacterial blooms and accumulation of microcystins in aquatic animals [1, 7].

According to our results a spatial distribution of microcystins in the same area, in different seasons was detected. This could be explained by the different levels of enrichment in the coastal zones by the runoffs of the rivers, carrying nutrients. Axios river is near Kavoura in Chalastra, Aliakmonas river is near Makrigialos in Pieria and Loudias river is near Klidi in Imathia. Their annual runoffs are 158 up to 279 m^3^ s^−1^, 73 up to 137 m^3^ s^−1^and 5 to 10 m^3^ s^−1^, respectively. Large agriculture activity is located in the adjacent areas with the transferred sediments, which are rich in nitrates and phosphates, are up to 500 t km^− 2^ [62]. The highest amounts of runoffs in Kavoura, Imathia and the estuaries are observed during spring [62], while in Makrigialos the lowest are observed during summer [63].

Regarding the comparison between ELISA and LC-Orbitrap HRMS, our results indicate an overestimation by ELISA. In particular, during spring the samples above 1 ng g^−1^ were 24 by ELISA and 7 by LC-Orbitrap HRMS, in summer 12 and 4, respectively, and in autumn 16 and 4, respectively. Moreover, the concentrations obtained by ELISA were 6 to even 22 times higher. According to the manufacturer’s instructions, the microcystins standards in ELISA were MC-LR, MC-RR, MC-YR, MC-LF, MC-LW, and the desmethylated [D-Asp3] MC-RR and [Dha7] MC-LR. On the other hand, the LC-Orbitrap HRMS analysis focused on the most common microcystins MC-LR, MC-RR and MC-YR. Although ELISA is considered a suitable screening method due to its high sensitivity, detection of multi analogues of microcystins, rapid results, low cost and lack of ethical issues [26–29], false positive results due to matrix effects cannot be excluded. Similar observations have also been reported in other studies. In a study in bovine drinking water, *Microcystis aeruginosa* cells were added at the abundance of 10^5^ cells mL^−1^ for 28 days. The microcystins concentrations in the liver were 0.92 µg MC-LR equivalents per g fresh weight measured by ELISA, although no toxins were detected by HPLC/GC-MS [64]. It was concluded that the levels were 1000 times higher due to matrix effect. In another study during an expansion of a cyanobacterial bloom in farms of *M. galloprovincialis* in Adriatic Sea [60], the MC-LR equivalents were 256 ng g^−1^ (ELISA) in mussels’ tissues, although only the desmethylated derivative desMe-MC-RR was confirmed by LC/ESI-Q-ToF-MS/MS at the level of 39 ng g^−1^. In other mussel samples an overestimation was also observed (1.5 to 6.5 times higher). It was mentioned that ELISA is a useful screening tool, but results should be confirmed by chromatographic analytical methods tandem to mass spectrometry. In another study in McMurdo Ice Shelf station in Antarctica, researchers from New Zealand examined water samples and cyanobacterial mats [65]. The concentrations of microcystins were up to 8 times higher by ELISA than LC-MS. This overestimation could lead to unnecessary bans in drinking water, so the results should be confirmed by mass spectrometry. A similar study evaluated the results obtained by ELISA and LC-MS/MS [66]. It was proposed that ELISA could be used for screening, since it provides rapid results. Still, it was reported that the non linear calibration curve could lead to false positive results, since small differences in absorbances might be translated into big concentration differences. The same authors report that LC-MS/MS provides higher specificity and sensitivity and concluded that in any case the abundance of cyanobacteria and the water treatment should be considered. Also, microcystins concentrations have been assessed in nontoxic cyanobacterial food supplements, peals and capsules [67]. It was reported that no statistically significant differences were observed in capsules, since the concentrations were 43 to 410 ng per capsule and 40 to 425 ng per capsule by ELISA and LC/MS, respectively. In peals the levels by ELISA were lower, 200 to 960 ng per peal, when by LC/MS they were 280 to 1310 ng per peal. So, an underestimation at the level of 27% was observed by ELISA. This was explained by the different sensitivity of the LC-MS method in some microcystins analogues. Finally, in a recent study [68] water samples from 31 water ecosystems in Michigan, USA, were examined by both methods, ELISA and LC-MS for microcystins, during July, August, September and October, when cyanobacterial blooms often occur. It was reported that no statistically significant differences were observed in July and August. On the other hand, an overestimation by ELISA was noticed in September and October. This was attributed to cross-reactivity with microcystins’ degradation products and to the quantification using nonlinear calibration curve.

## Conclusions

In our study microcystin-RR and microcystin-LR were detected for first time in mussel *M. galloprovincialis*, harvested from farms in Thermaikos gulf, in Central Macedonia, in Greece. The presence of potentially toxic cyanobacteria in those areas was confirmed molecularly. Bioaccumulation in digestive gland was observed, while the concentrations in muscles were extremely low. Samples with levels above 1 ng g^−1^ were observed mostly during spring, confirming the seasonal distribution of microcystins. Moreover, a spatial distribution among sampling areas was noticed, explained by the fluctuations of the rivers runoffs and the nutrients enrichments. The analysis by ELISA and LC-Orbitrap HRMS, revealed overestimation of the first method. In any case, the use of ELISA as screening method is recommended to be followed by chromatographic analytical methods and mass spectrometry as confirmatory methods.

## Data Availability

The datasets used and/or analyzed during the current study are available from the corresponding author on reasonable request.
